# Patient-Reported Quality of Communication Skills in the Clinical Workplace for Clinicians Learning Medical Spanish

**DOI:** 10.7759/cureus.22222

**Published:** 2022-02-15

**Authors:** Pilar Ortega, Santiago Avila, Yoon Soo Park

**Affiliations:** 1 Medical Education, University of Illinois College of Medicine at Chicago, Chicago, USA; 2 School of Medicine, The University of Chicago Pritzker School of Medicine, Chicago, USA

**Keywords:** limited english proficiency, patient-centered communication, hispanic/latino healthcare, communication skills, medical spanish, language-appropriate healthcare, language-concordant healthcare, interprofessional education

## Abstract

Introduction

Patient-clinician communication is a key factor in patient satisfaction with care. Clinicians take medical language courses to improve communication with linguistically diverse populations, yet little is known about how patients perceive clinicians’ skills.

Methods

We designed a prospective, comparative survey study of patient perception of clinician communication using a convenience sampling of health professionals enrolled in an interprofessional medical Spanish course. We analyzed the patient-reported quality of communication skills from 214 clinical encounters and self-evaluations of 18 clinicians with Spanish- and English-speaking patients.

Results

Communication scores were lower for Spanish vs. English encounters as reported by both patients and clinicians (*p*<0.001). Clinician-reported scores were lower than patient-reported scores in Spanish encounters (9.05±0.23 vs. 8.05±0.23; *p*<0.001), whereas there was no difference in English encounters (11.17±0.15 vs. 11.35±0.19; *p*=0.914). The effect of language remained significant (*p*<0.001) when controlling for medical setting and complexity.

Conclusion

Spanish-speaking patients report lower-quality communication from clinicians learning Spanish than do English-speaking patients. Incorporating and further evaluating patient perceptions of clinician Spanish communication skills may improve language-appropriate healthcare and clinician education.

## Introduction

Of the over 25 million individuals in the United States with limited English proficiency (LEP), approximately 16 million are Spanish speakers [1]. By contrast, the number of Spanish-speaking clinicians has remained stagnant, resulting in a progressively worsening language concordance deficit [2]. Many healthcare professionals, including those who self-identify as Hispanic/Latinx and those who do not, report having some Spanish language skills and using their inpatient care, sometimes foregoing professional interpreters [3]. However, the criteria by which these clinicians decide to use their language skills to practice medicine in Spanish are unclear and variable due to the lack of standardized guidelines, validated assessment methodologies, and training [4].

Linguistically and culturally appropriate communication with Spanish-speaking patients

Patient-clinician communication is a key factor in patient satisfaction with care [5]. Inadequate communication contributes to U.S. Hispanic/Latinx patients with LEP experiencing poor-quality healthcare, worse outcomes, and more adverse events [6-7]. Language discordance results in inequitable healthcare delivery and lower patient satisfaction [8-9]. Data show that culturally competent and language-concordant care improves patient satisfaction when compared to interpreter-mediated language-discordant encounters [10-12]. Perceived clinician cultural sensitivity also modulates patient trust and satisfaction and, in turn, drives medication adherence in Hispanic/Latinx patients [13]. While some studies suggest that patient satisfaction is higher among first-generation Hispanic/Latinx immigrants, a phenomenon attributed to the “Happy Migrant Effect” [14], others suggest that Hispanic/Latinx patients as a whole may be less satisfied with their healthcare services [15]. These complex factors should be considered when evaluating patient satisfaction data. For example, immigrant patients may be less likely to express negative perceptions regarding the healthcare they receive due to fear of potential repercussions. One study found that insurance, educational level, and acculturation significantly influenced patient satisfaction measures in Hispanic/Latinx patients [16]. Strong family and social support and access to mainstream media in individuals’ preferred language may also modulate trust in health information sources such as clinicians [17].

Similarly, the use of highly technical language in health education materials negatively affects the perceived expertise of the author and the credibility of the information [18]. Overuse of medical jargon is a potential consequence of training clinicians in medical Spanish or other languages. Learning programs that focus exclusively on vocabulary building without teaching comprehension or patient-centered skills may inadvertently increase communication problems by giving clinicians a sense that they are adequately communicating medical information even when that is not the case [19].

Measuring Spanish-speaking patient perceptions of care

The data on Spanish-speaking patients' perception of healthcare services is limited. Without appropriately gauging patient perception and satisfaction, medical institutions should not equitably allocate resources to improving services for underserved communities, for example, patients with non-dominant language preferences. Patients with LEP are also less likely to be included in healthcare quality improvement or clinical research efforts due to the unavailability of translated forms, bilingual research staff, or medical interpreters [20], resulting in insufficient representation.

Some studies have evaluated the effect of language concordance on patient satisfaction in clinical settings, for example, by using the interpersonal processes of care (IPC) in diverse populations' instrument [10,21]. The IPC instrument focuses on communication in primary care settings, asking patients to gauge the effectiveness of communication with their outpatient clinicians for the last six months [21]. To be useful for clinicians learning Spanish, who may represent a wide range of healthcare roles including urgent/emergency care, patient feedback regarding communication effectiveness should be sought immediately after the encounter, rather than after six months. Immediate feedback may allow clinicians to better understand their communication strengths and weaknesses while they are taking a course to refine their learning objectives and focused areas of study.

Despite the recent proliferation of programs to increase language concordance by teaching clinicians Spanish skills [22], no published report addressed the patient's perception of the communication skills of providers learning Spanish. Recent literature emphasizes the need to teach learners to accurately and progressively self-assess their linguistic limitations to ensure safe use of Spanish skills [19]. Self-assessment is an important part of training clinicians to use their language skills responsibly, but experts recommend that self-assessment alone is not sufficient to determine readiness to use language skills in clinical practice [3,23] but they should also be complemented by feedback from trained observers. Moreover, data show that medical students with some Spanish skills use the language in patient care out of perceived necessity or urgency [24], even though they might think that their skills are insufficient. As a result, Spanish-speaking patients with LEP are often exposed to clinicians-in-training who use medical Spanish to communicate with them. These patients have an important perspective to share but may not feel comfortable providing negative feedback to well-intentioned clinicians, who are trying their best to communicate with them in their preferred language.

The effects of language educational interventions on patient perception or satisfaction are not well studied. To address this gap, this study examines how Spanish-speaking patients perceive the communication skills of clinicians enrolled in an interprofessional education (IPE) medical Spanish course. Specifically, we aimed to (a) explore trends in communication skill ratings for clinicians’ Spanish vs. English encounters and (b) compare patients’ assessment of the quality of communication to the clinicians’ self-assessment for the same medical encounter.

## Materials and methods

We designed a prospective, cross-sectional study of patient perception of clinician communication using a convenience sampling of health professionals enrolled in an IPE medical Spanish course.

Participants

As part of an eight-week IPE medical Spanish continuing medical education (CME) course offered at an urban, comprehensive medical center, clinician learners participated in a study to evaluate patient perceptions of their language skills. The course targeted staff members with a pre-existing, self-reported Spanish proficiency of low-to-intermediate or higher, as recommended for medical Spanish courses [19]. The course recommended, but did not require, a minimum Spanish level of “fair” (low-to-intermediate level) or above on the Interagency Language Roundtable (ILR) scale, modified for healthcare [3].

Interprofessional educational curriculum

The IPE medical Spanish course provided up to 12 CME credits to learners and was implemented in two 8-week sessions from April to May 2017 and August to September 2017. The curriculum emphasized both theoretical and practical Spanish clinician-patient communication skill development. The course’s principal learning objective was for learners to improve their patient-centered Spanish communication skills. Using this main objective as a starting point, learners worked with the instructor to create more specific, individualized learning objectives depending on their Spanish level and job responsibilities. For example, novice-level learners focused on introducing themselves, building rapport, and asking basic questions while waiting for the medical interpreter, whereas advanced-level learners focused on conducting a complete medical interview, explaining diagnoses, and discussing treatment plans with patients.

Communication survey instrument

We designed an anonymous survey (“communication survey”) to assess perceptions of clinician communication immediately following medical encounters taking place while the clinicians were enrolled in the medical Spanish course. The brief survey contained three Likert-style questions (rated on a 5-point scale) to assess the patient’s perceptions and clinician’s self-perception of the clinician’s communication skills (see Appendix).

We designed the communication survey by modifying existing questions from medical Spanish standardized patient (SP) feedback checklists [25] that had been previously developed by a medical educator who is a member of the research team. These checklists had been previously piloted and tested for use by SPs who participated in medical Spanish simulated encounters [25]. The SPs who had previously tested the instrument self-identified as Hispanic/Latinx were of Mexican heritage and preferentially reported using Spanish for healthcare communication. This approach ensured that survey questions would be relevant to understanding clinicians’ progress in achieving their Spanish learning objectives and that the questions would be appropriate for patients with a Spanish language preference. Given that SPs receive training before using the questionnaire and the same would not be true of patients during real clinical encounters, it was important to further modify the questionnaire for ease of use in these less-predictable scenarios and to avoid overburdening patients. Thus, we selected questions that focused on patient-centered communication skills, including whether the clinician understood the patient’s story, whether the patient understood the clinician’s questions, and whether the patient understood the clinician’s explanations about diagnosis or treatment.

To ensure that our survey reflected evidence-based literature on Spanish-speaking patient satisfaction with communication, we compared our questions to those of the IPC instrument as applied by Fernandez et al. [10] in evaluating language-concordant care in outpatient primary care settings and noted that our questions addressed several of the same areas of clinician communication: general clarity, elicitation and responsiveness to patient problems, and explanation of condition/processes of care. We then piloted the modified Spanish questions with two native Spanish speakers and the English questions with two native English speakers and used their feedback to further revise the instrument for clarity and simplicity before patient use.

All clinician surveys were in English (the clinicians’ preferred language) and contained three additional items about the setting of the encounter, whether it was a first-time or follow-up encounter, and the level of medical complexity. The patient surveys were available in Spanish or English.

Data collection

During the IPE medical Spanish course, participating clinicians provided communication surveys to individual patients in their usual clinical practice immediately following Spanish or English clinical encounters for which they determined they did not need a medical interpreter. Each clinician solicited evaluations from both Spanish- and English-speaking patients. Patients received evaluations in an envelope and were invited to voluntarily complete the anonymous survey, seal it in the envelope provided, and return the sealed envelope to the nurses’ station to encourage honest feedback without fear of retribution or disclosure to the clinician. Patients were assured that the clinician would not know whether they completed the survey, that their responses or lack of participation would not affect their care, and that the surveys would not be traceable to individual respondents. The surveys did not collect any protected health information.

To ensure that clinicians did not feel obligated or pressured to forego using a medical interpreter when needed, the course directors invited, but did not require, clinician learners to participate in the communication survey study. In addition, if they chose to participate, the score they achieved did not impact their grade performance or course credit. We pre-assigned a unique number to each clinical encounter to track the corresponding patient and clinician surveys. The unique encounter numbers matched clinician survey responses (i.e., self-evaluations) with patient surveys. We collected all data between April and September 2017. The Advocate Health Institutional Review Board determined this study to meet the criteria for exemption on February 11, 2017.

Analysis

We calculated communication survey scores (ranging from a minimum total score of 3 to 12) by summing responses from each survey item. We compared the reported quality of communication for the same clinicians by patients with Spanish and English language preferences to control for other variables besides language that may affect whether a clinician is perceived as a good communicator. We also analyzed the data for the effect of potential confounding factors including medical encounter complexity, whether the encounter was a first-time or follow-up encounter, and setting of inpatient, outpatient, or emergency department. We did not evaluate self-reported clinician language proficiency as a confounding variable since all clinician learners in our course were at a similar proficiency level. We conducted data compilation and analyses using Stata Statistical Software Release 16 (StataCorp LLC, College Station, TX). We used *t*-tests to compare means between continuous variables (i.e., mean communication scores), Chi-squared tests to test the differences between categorical variables (i.e., clinician characteristics, encounter type, encounter setting, and language), and Kruskal-Wallis tests to examine the differences between ordinal variables (i.e., medical complexity). To examine simultaneous effects of factors accounting for patient-level clustering effects, we used mixed-effects linear regression, specifying random intercepts at the clinician level.

## Results

Descriptive statistics

Fifty percent (18 of 36) of clinicians enrolled in the IPE medical Spanish course participated in the patient perception study. Of these 18 clinicians, 17 (94%) provided complete demographic information and 17 (94%) provided complete Spanish proficiency information. Table 1 presents the descriptive characteristics of the clinician participants, including their pre-course Spanish proficiency level and experience. The clinicians enrolled in the course were primarily physicians and nurses, and a majority (11 of 17; 65%) reported a low-to-intermediate pre-course Spanish proficiency level. Communication surveys were completed for 214 patient encounters. Table 1 also presents the descriptive characteristics of all encounters that were evaluated in the study.

**Table 1:** Descriptive characteristics of clinician participants (n=18) in the IPE medical Spanish course and their scored patient encounters (n=214); Column % Abbreviations: IPE, interprofessional education; ED, emergency department. ^a^Among the 18 clinicians, all provided gender, profession, and race data. One student did not provide data for the number of years in the current position, two students did not provide prior Spanish experience, and one student did not provide the baseline level of general Spanish proficiency. ^b^Chi-square tests for prior Spanish experience types were conducted individually (per experience type, by row), whereas all other Chi-square tests were conducted across all characteristic subtypes. ^c^*p* < 0.001. ^d^*p* < 0.01. ^e^*p* < 0.05.

Clinician Characteristics		Clinicians (*n*=18)^a^	Patient Encounters^b^ (*n*=214)									
			Encounter Language		Encounter Type		Encounter Setting			Medical Complexity		
			Spanish	English	First-time	Follow-up	Inpatient	Outpatient	ED	Simple	Moderate	High
Gender	Female	78	40	30	49	20	9^c^	34^c^	20^c^	21^c^	29^c^	18^c^
	Male	22	16	16	22	10	20^ c^	7^c^	10^c^	16^c^	16^c^	1^c^
Race/ethnicity	White	67	33	29	41	16	9^c^	27^c^	26^c^	14^c^	31^c^	17^c^
	Hispanic/Latinx	11	12	12	17	9	15^c^	10^c^	1^c^	17^c^	7^c^	0^c^
	Black/African American	1	4	2	5	0	0^c^	2^c^	0^c^	5^c^	1^c^	0^c^
	Asian	11	2	1	4	1	0^c^	1^c^	3^c^	1^c^	2^c^	1^c^
	Other	6	3	2	3	3	4^c^	1^c^	0^c^	1^c^	4^c^	0^c^
Profession	Resident physician	17	4	3	6	3	4^c^	1^c^	0^c^	1^c^	6^c^	1^c^
	Registered nurse	73	39	31	47	22	9^c^	34^c^	31^c^	21^c^	32^c^	18^c^
	Radiation therapist	6	4	2	5	0	0^c^	2^c^	0^c^	5^c^	1^c^	0^c^
	Social worker	6	8	10	13	4	15^c^	4^c^	0^c^	10^c^	7^c^	0^c^
Years in current position	<3 years	47	16	11	8^d^	10^d^	5^c^	5^c^	12^c^	8^c^	14^c^	4^c^
	3-6 years	29	15	14	23^d^	8^d^	2^c^	11^c^	19^c^	7^c^	12^c^	10^c^
	6-10 years	12	11	10	22^d^	4^d^	8^c^	15^c^	0^c^	2^c^	14^c^	6^c^
	>10 years	12	12	12	20^d^	4^d^	17^c^	7^c^	0^c^	16^c^	8^c^	0^c^
Prior Spanish experience	Grade-school classes	19	10	5	8	5	2^c^	2^c^	11^c^	6	7	2
	High-school classes	69	43	40	62^e^	26^e^	29^c^	35^c^	23^c^	23	42	17
	Basic classes in college	19	5	5	5	1	0^e^	5^e^	5^e^	4	4	2
	Study abroad	13	7	7	11	1	0^c^	4^c^	11^c^	5	7	1
	Medical Spanish class	6	1	2	2	0	0	0	0	1	1	1
	Online classes	6	2	2	5	0	0^d^	0^d^	4^d^	0	3	1
	None	6	4	2	6	0	0	2	0	5^c^	1^c^	0^c^
General Spanish proficiency	0: None	6	4	2	6^d^	0^d^	0^c^	2^c^	0^c^	5^c^	1^c^	0^c^
	1: Beginner	24	13	10	10	10	6^c^	11^c^	7^c^	3^c^	12^c^	9^c^
	2: Low intermediate	65	32	29	51	17	23^c^	28^c^	14^c^	24^c^	29^c^	9^c^
	3: High intermediate	6	5	5	6	1	0^c^	1^c^	11^c^	4^c^	5^c^	0^c^
	4: Advanced	0	0	0	0	0	0^c^	0^c^	0^c^	0^c^	0^c^	0^c^
	5: Native/near-native	0	0	0	0	0	0^c^	0^c^	0^c^	0^c^	0^c^	0^c^

Each participating clinician provided communication surveys for an average of 11.9 patient encounters (range: 1-36). Patient responses were submitted for 212 communication surveys (99%). Overall, 208 encounters (97%) had complete data (both the clinician and patient surveys were completed). Clinical characteristics were not provided for all encounters (encounter type missing in 66; setting, 31; complexity, 3). Table 2 presents the communication scores for all evaluated patient encounters by encounter type, setting, complexity, and language. The distribution of clinical characteristics of encounter type, setting, and medical complexity did not significantly differ in Spanish vs. English encounters.

**Table 2:** Patient-reported and clinician self-reported communication survey scores for clinical encounters (n=214) during the IPE medical Spanish course by encounter type, setting, complexity, and language Abbreviations: IPE, interprofessional education; SE, standard error; ED, emergency department.​​​​​​ ​^a^Pearson’s Chi-squared test used for between-group comparisons of categorical variables (encounter type, encounter setting, and language). ^b^Kruskal-Wallis test used for between-group comparisons of ordinal variables (medical complexity).

	Encounter Characteristic	Patient Encounters (column %)	Patient-Reported Scores (SE)	*p*-Value^a,b^	Clinician Self-Reported Scores (SE)	*p*-Value^a,b^
Encounter type (*n*=148)	First-time	105 (71%)	9.91 (0.24)	0.858	9.58 (0.29)	0.998
	Follow-up	43 (29%)	9.83 (0.36)		9.58 (0.44)	
Encounter setting (*n*=183)	Inpatient	52 (28%)	10.69 (0.28)	0.024	10.10 (0.38)	0.206
	Outpatient	75 (41%)	9.84 (0.28)		9.47 (0.34)	
	ED	56 (31%)	9.64 (0.32)		9.33 (0.32)	
Medical complexity (*n*=211)	Simple	78 (37%)	10.65 (0.20)	0.006	10.59 (0.25)	<0.001
	Moderate	95 (45%)	9.79 (0.25)		9.23 (0.27)	
	High	38 (18%)	9.16 (0.46)		8.27 (0.55)	
Language (*n*=214)	Spanish	116 (54%)	9.04 (0.23)	<0.001	8.05 (0.23)	<0.001
	English	98 (46%)	11.17 (0.15)		11.36 (0.19)	

Survey reliability analysis

The clinician survey had a Cronbach’s alpha of 0.98 and 0.90, whereas the patient survey had a Cronbach’s alpha of 0.87 and 0.93 in English and Spanish, respectively.

Spanish vs. English encounters

**Figure 1:**
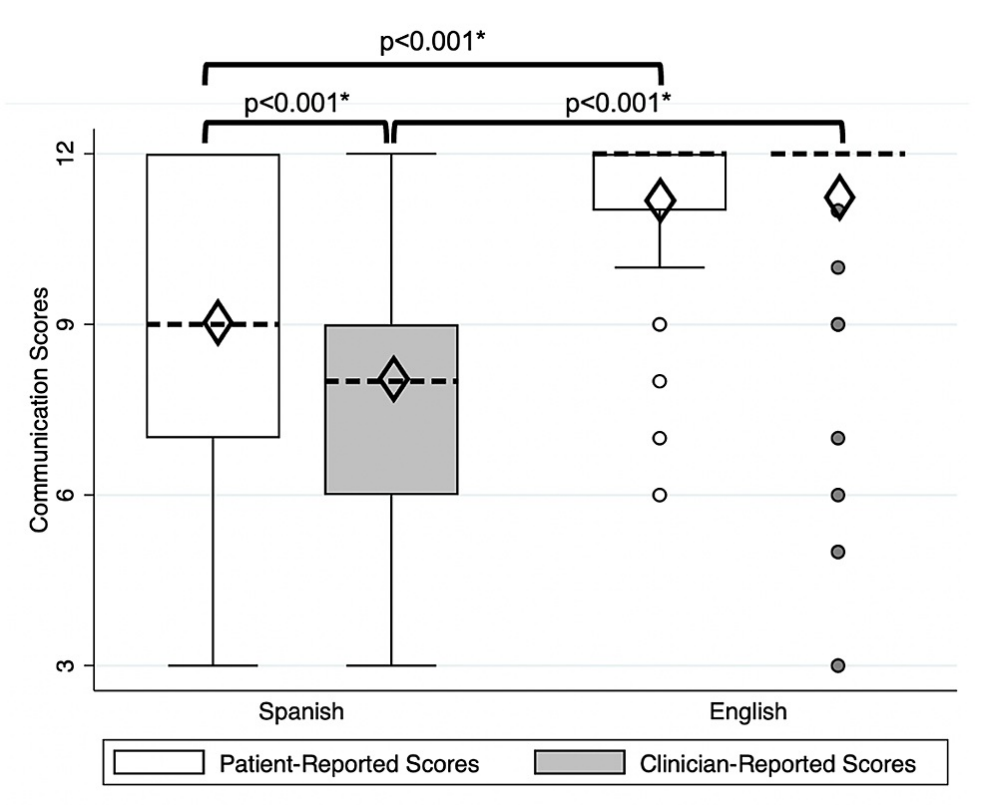
Patient- and clinician-reported communication scores for Spanish (n=116) and English (n=98) clinical encounters during the IPE medical Spanish course Boxes represent median, interquartile ranges with whiskers indicate the highest and lowest values within 1.5 times the interquartile range, and dots represent outlying data. Dashed lines represent median scores, and diamonds represent mean scores. Brackets represent significant relationships between different scores. Abbreviations: IPE, interprofessional education. *Paired *t*-test of means.

Across patient encounters, communication survey scores were significantly lower for Spanish encounters as opposed to English encounters as reported by both patients and clinicians. Median patient communication scores were 9 [interquartile range (IQR): 7-12] for encounters in Spanish and 12 (IQR: 11-12) for encounters in English (*p*<0.001). Median clinician self-reported communication scores were 8 (IQR: 6-9) for encounters in Spanish and 12 (IQR: 12-12) for encounters in English (*p*<0.001), as illustrated in Figure 1. The clinician communication scores in English displayed narrow variance. These data highlight that clinician communication during Spanish encounters was consistently scored lower than their communication during English encounters and that there was a wider range of scores in Spanish performance compared to English encounters. The English encounters’ results yielded a narrower IQR compared to Spanish, suggesting greater clinician communication consistency in English compared to Spanish. 

Patient-reported vs. clinician self-reported communication scores

Overall, patient-reported communication scores were significantly higher than self-reported scores by clinicians (10.01±0.16 vs. 9.54±0.19; *p*<0.001). For encounters in Spanish, patient-reported scores were significantly higher than clinician self-reported scores (9.05±0.23 vs. 8.05±0.23; *p*<0.001). Conversely, for English encounters, there was no significant difference between patient-reported and clinician self-reported scores (11.17±0.15 vs. 11.35±0.19; *p*=0.914).

Analysis of the data for the effect of potentially confounding variables demonstrated that the outpatient setting did have a significant impact on patient-reported scores, whereas both the outpatient setting and increasing medical complexity had a significant impact on clinician-reported scores. Specifically, clinicians self-reported lower communication scores were reported as more complex (*p*<0.001). Also, patient- and clinician-reported communication scores were significantly lower in the outpatient setting when compared to the inpatient setting (*p*=0.025; *p*=0.024). However, even when we controlled for the variables of encounter type, setting, and medical complexity, both patient-reported and clinician self-reported communication scores were still significantly lower for Spanish vs. English encounters (all *p*<0.001). There was significant variability in patient-reported scores and clinician self-reported scores, depending on the patient seen, as noted by the sizeable random-effect variability estimates. Table 3 presents the data that includes confounding factors. First-time or follow-up encounters were not significantly different.

**Table 3:** Factors affecting patient-reported and clinician self-reported communication survey scores: mixed-effects regression Abbreviations: SE, standard error. SD, standard deviation of the random-intercept parameter. ^a^*p*<0.001. ^b^*p*<0.01. ^c^*p*<0.05.

Variable		Patient-Reported Scores		Clinician Self-Reported Scores	
		Coefficient (SE)	*p*-Value	Coefficient (SE)	*p*-Value
Language	Spanish	Reference		Reference	
	English	2.04 (0.79)	0.009^b^	3.10 (0.92)	0.001^b^
Encounter type	First-time	Reference		Reference	
	Follow-up	0.27 (0.21)	0.193	0.65 (0.55)	0.236
Encounter setting	Inpatient	Reference		Reference	
	Outpatient	-0.50 (0.52)	0.337	-0.75 (0.36)	0.037^c^
	Emergency department	-0.64 (0.65)	0.328	-0.41 (0.71)	0.558
Medical complexity	Simple	Reference		Reference	
	Moderate	-0.16 (0.22)	0.47	-0.23 (0.23)	0.306
Random effect: SD estimate (SE)		1.51 (0.33)	<0.001^a^	1.86 (0.67)	<0.001^a^

## Discussion

Our study examines the patient-reported quality of communication during health encounters with clinicians who are medical Spanish learners. The study is contextualized in a healthcare system in which English is the dominant professional language, yet has a growing Spanish-speaking patient population. To our knowledge, this is the first study to report patient perceptions of clinicians’ language skills in clinical care with Spanish-speaking LEP patients while enrolled in a Spanish course. Overall, both patient-reported and clinician self-reported communication scores were lower in Spanish encounters than in English encounters. It is understandable that English-speaking clinicians learning Spanish will not have equivalent communication skills in Spanish as they do in English. For this reason, they need guidance in the progressive mastery of their Spanish skills [23]. While prior work has centered on faculty and SP feedback [25], LEP patients represent another important source of feedback, particularly for clinicians already in practice working toward improved communication with specific linguistic groups. As the person who stands the most to gain (or to lose) from the quality of health communication, patients are the most knowledgeable individuals in assessing their own sense of satisfaction with clinician communication. Patients are also the most appropriate judges of their individual language preferences, including regional variants in pronunciation, accent, and terminology, which may vary within languages. Requesting feedback from LEP patients regarding clinicians’ use of non-dominant language skills may help inform training and assessment strategies for health professionals that prioritize patient needs.

The use of Spanish by clinicians with partial Spanish skills in a medical encounter was associated with significantly lower scores in both patient and clinician communication ratings when compared to those same clinicians’ English encounters. These differences remained when we controlled for encounter type, setting, and medical complexity. Follow-up encounters were not associated with improved patient- or clinician-reported communication scores, suggesting that established relationships with patients did not alter the perception of language skills.

Importantly, patient perception of communication skills may depend not only on the linguistic abilities of the clinician but also on their overall interpersonal and communication skills as situated within the sociocultural context of the patient [26]. For example, some interpersonal behaviors (e.g., eye contact, physical contact, specific gestures) may be perceived differently across cultures and may influence trust. Similarly, clinicians should adjust the register (i.e., the degree of formality and complexity of their speech) depending on real-time feedback regarding patient comprehension, which may, in turn, differ based on age, level of education, or other factors. Interpersonal communication skills, in combination with the patient’s cultural lens, can impact not only satisfaction/trust [8] but also health outcomes [27]. While our study did not investigate the reasons for which patients gave clinicians specific communication scores, the finding that communication scores differed in Spanish vs. English encounters highlights the need for medical Spanish education to go beyond teaching vocabulary alone [28]. In addition to language, medical Spanish courses should address the multiple layers of communication skills needed for the effective care of Spanish-speaking patients, including trust and rapport-building, interpersonal behaviors, intonation, and cultural notions of politeness and respect.

Educational implications

The overall lower communication scores for Spanish encounters suggest that an IPE medical Spanish course for working clinicians may need to be accompanied by other progressive, longitudinal interventions to attain Spanish communication skills that are within a comparable range to English. It is important to note that the majority of clinicians enrolled in the course had a low-to-intermediate Spanish proficiency, and some had a lower skill level than is recommended for participating in medical Spanish courses. The low pre-course Spanish proficiency levels of the majority of clinicians enrolled in the study are certainly related to their lower scores in Spanish encounters compared to English, supporting the recommendation that medical Spanish courses may be more suitable for speakers at intermediate or higher proficiency levels [19]. This study also adds to the literature on medical Spanish education by reporting on an IPE course, whereas most previous studies have focused on courses within a single health profession only (e.g., Spanish for doctors, Spanish for nurses, Spanish for pharmacists, etc.). Incorporating strategies to solicit patient feedback about learner language skills may be helpful to learners at multiple points of their interprofessional medical Spanish training and may increase clinicians’ progressive awareness of their communication skills and limitations.

Clinical practice implications

Our study shows that clinicians choose to use Spanish in patient care despite knowing that their skills are limited, adding to similar prior data from medical school settings [24]. The lower Spanish communication scores of clinicians learning medical Spanish (compared to their English performance) underscore the longitudinal nature of language acquisition. Additionally, it highlights the critical need to make professional medical interpreters and other high-quality language resources readily available to clinicians (including medical Spanish learners) to support their communication needs with linguistic groups. Clinicians should not be expected to achieve mastery of communication skills in a non-dominant language during a single course.

While clinicians should be encouraged to continue their medical Spanish learning, they should also be encouraged to responsibly work with professional interpreters while they acquire mastery of skills. However, encouragement alone may not be effective when hospital systems do not make such language services readily accessible to staff [29]. Additionally, increasing access to medical Spanish curricula at the undergraduate and health profession school levels as part of communication skills training may help clinicians start on their longitudinal language learning trajectory earlier and may yield better patient satisfaction results. The act of requesting feedback from patients and reflecting on their own performance may help clinicians build greater awareness of self-limitations than they had previously.

Interestingly, our data show that clinician self-reported communication scores were lower than those reported by patients in Spanish encounters. In other words, patients reported Spanish communication by clinicians more positively than the clinicians did themselves. This finding suggests that patients may be more “forgiving” of clinicians who are trying their best to speak to patients in their preferred language, whereas clinicians may be more self-critical. Socioculturally, Spanish speakers may also be unable to provide negative evaluations of healthcare staff despite assurances regarding anonymity. Moreover, a recent systematic review of patient and physician communication perspectives suggests that when evaluating the quality of communication, patients and clinicians may prioritize different facets of communication [30]. These inherent differences in perspective may partially drive the differences we report in communication evaluations of Spanish-speaking patients and further support the importance of obtaining feedback from multiple stakeholders. In our study, the differences between clinicians and patients were only detected in Spanish encounters, suggesting that further study on patient perception of healthcare communication may be particularly critical in linguistically diverse populations.

Limitations and future study

This study has some limitations. As an exploratory study, we opted to provide a brief survey that was limited in scope to increase the ease of implementation and to not over-burden linguistic minority patients or the clinicians enrolled in a continuing education course. Future studies could further refine our communications survey by incorporating additional questions from the IPC instrument [10] to obtain more nuanced information about specific components of clinician communication performance in linguistically diverse settings.

Additionally, clinicians selected which patients would receive a survey, which could result in selection bias. While clinicians were instructed to seek evaluations from any patient encounter conducted either in Spanish or in English without an interpreter, they might have asked patients to complete the survey if the encounter went particularly well or forgone selecting patients if they felt the encounter went poorly. The results of this study also underscore substantial variability at the patient level (as noted in the sizeable random-effects variance) that can influence outcomes related to communication skills. Future studies can be designed to examine factors contributing to the heterogeneity at the patient level, possibly considering a mix of patient populations to facilitate broader understanding. For example, factors such as age, comorbidities, illness severity, level of education/health literacy, and nationality or ancestry of origin could potentially influence how clinician communication skills are perceived. These factors may facilitate deriving more stable and consistent effect sizes with sufficient sampling and power, based on the findings of this work.

Collecting data from clinicians with a wider breadth of Spanish levels, such as advanced speakers, would further add to a more nuanced understanding of the patient perception of clinician communication skills. Future studies should evaluate whether there are changes to patient perception of clinician language skills before and after IPE interventions and could compare patient perspectives to feedback provided by trained raters such as faculty and SPs.

Lessons learned

We summarize the following three key lessons learned from our study: (1) Clinicians with limited Spanish skills are still using those skills with patients, despite recognizing that the skills are of lower quality than their English communication, suggesting that there is a need to create standards for the use of non-dominant language skills in healthcare. (2) Health systems should provide clear guidance for clinicians with some multilingual skills to help them accurately determine when they should use their skills in patient care and when they should use professional interpreters. (3) Patient perception of clinicians’ communication skills may be an important way for clinicians to gain insights into their skills and limitations, identify areas for improvement, and ensure that educational curricula prioritize patient perspectives.

## Conclusions

Our study adds to the limited literature on IPE medical Spanish courses and on patient perceptions of clinicians’ use of healthcare Spanish. Spanish-speaking patients perceive clinicians with partial Spanish language skills as providing less effective health communication during Spanish medical encounters when compared to how well those same clinicians are perceived to communicate by English-speaking patients. This work demonstrates that it is feasible to engage Spanish-speaking patients in providing their opinion about clinician communication, and this approach may inform future strategies for improving healthcare for LEP populations through clinician IPE. As the U.S. population of Spanish speakers continues to rise, accounting for the patient perception of clinician Spanish communication skills is a critical element in improving language-appropriate health professional education and reducing language-based healthcare inequities.

## Appendices

**Table 4:** Clinician and Patient Communication Surveys

Survey Respondent	Question Item	Response scale
Clinician communication survey	1. I was able to understand the patient	Very well, well, fairly, poorly
	2. The patient was able to understand my questions	Very well, well, fairly, poorly
	3. The patient was able to understand my explanations	Very well, well, fairly, poorly
	4. How medically complicated would you rate this encounter to be (regardless of language).	Simple, moderate, high complexity
	5. Encounter characteristics: Inpatient, outpatient, emergency department, first-time encounter, follow-up encounter, other	Select all that apply
Patient communication survey (English)	1. I felt that this provider (e.g., doctor/nurse) understood me	Very well, well, fairly, poorly
	2. I was able to understand the questions that this provider asked me	Very well, well, fairly, poorly
	3. I was able to understand the explanations that this provider gave me	Very well, well, fairly, poorly
Patient communication survey (Spanish)	1. Sentí que este proveedor de salud (ej., doctor/a o enfermero/a) me entendió	Muy bien, Bien, Más o menos, Mal
	2. Yo pude entender las preguntas que me hacía este proveedor de salud	Muy bien, Bien, Más o menos, Mal
	3. Yo pude entender las explicaciones que me dio este proveedor de salud	Muy bien, Bien, Más o menos, Mal
